# Trendy: segmented regression analysis of expression dynamics in high-throughput ordered profiling experiments

**DOI:** 10.1186/s12859-018-2405-x

**Published:** 2018-10-16

**Authors:** Rhonda Bacher, Ning Leng, Li-Fang Chu, Zijian Ni, James A. Thomson, Christina Kendziorski, Ron Stewart

**Affiliations:** 1 Department of Biostatistics, University of Florida, Gainesville, FL USA; 2 Morgridge Institute for Research, Madison, WI USA; 3 Department of Statistics, University of Wisconsin-Madison, Madison, WI USA; 4 Department of Biostatistics and Medical Informatics, University of Wisconsin-Madison, Madison, WI USA

**Keywords:** Time-course, Gene expression, RNA-seq, Segmented regression, R package, Shiny

## Abstract

**Background:**

High-throughput expression profiling experiments with ordered conditions (e.g. time-course or spatial-course) are becoming more common for studying detailed differentiation processes or spatial patterns. Identifying dynamic changes at both the individual gene and whole transcriptome level can provide important insights about genes, pathways, and critical time points.

**Results:**

We present an R package, Trendy, which utilizes segmented regression models to simultaneously characterize each gene’s expression pattern and summarize overall dynamic activity in ordered condition experiments. For each gene, Trendy finds the optimal segmented regression model and provides the location and direction of dynamic changes in expression. We demonstrate the utility of Trendy to provide biologically relevant results on both microarray and RNA-sequencing (RNA-seq) datasets.

**Conclusions:**

Trendy is a flexible R package which characterizes gene-specific expression patterns and summarizes changes of global dynamics over ordered conditions. Trendy is freely available on Bioconductor with a full vignette at https://bioconductor.org/packages/release/bioc/html/Trendy.html.

**Electronic supplementary material:**

The online version of this article (10.1186/s12859-018-2405-x) contains supplementary material, which is available to authorized users.

## Background

High-throughput, transcriptome-wide expression profiling technologies such as microarrays and RNA-seq have become essential tools for advancing insights into biological systems. The power of these technologies can be further leveraged to study the dynamics of biological processes by profiling over ordered conditions such as time or space. In this article, we use the general term “time-course” to refer to any dynamically ordered condition and “gene” to any genomic feature (i.e. transcripts, exons).

Many methods for time-course experiments aim to identify differentially expressed genes between multi-series time-courses (e.g. two treatments monitored over time) [1–3]. A review of the statistical methods for multi-series experiments can be found in [4], and an evaluation of those methods is given in [5]. Alternatively, single-series time-course experiments, those where a single treatment is monitored over time, are also of biological interest. In these experiments, genes with dynamic expression patterns over time are identified, which can provide insight on regulatory genes [6] and reveal key transitional periods [7]. We focus our attention on single-series time-courses in this article.

Statistical methods for analyzing single-series time-course data have largely focused on clustering gene expression [8, 9]. These types of methods typically do not emphasize each gene’s individual expression path, instead they use the expression of each gene over time to form homogenous gene clusters which can then be used to construct regulatory networks or infer functional enrichment. FunPat [10] is one method focused on clustering genes, and rather than post-hoc enrichment analysis, it incorporates functional gene annotations directly into a model-based clustering framework.

EBSeq-HMM [11] was developed in part to address the deficiency in characterizing genes individually. It employs a hidden Markov model to classify each gene into distinct expression paths. Despite its utility, differences between time points may not be sufficiently detectable for extensive or densely sampled time-course experiments with subtle expression changes. Additionally, EBSeq-HMM is not suitable for long time-courses as the number of patterns it attempts to detect increases exponentially (the total number of patterns is 3^time points−1^).

Here we propose an approach we call Trendy which employs the method of segmented regression models to simultaneously characterize each gene’s expression pattern and summarize overall dynamic activity in single-series time-course experiments. For each gene, we fit a set of segmented regression models with varying numbers of breakpoints. Each breakpoint represents a dynamic change in the gene’s expression profile over time. A model selection step then identifies the model with the optimal number of breakpoints.

We define the top dynamic genes as those that are well-profiled based on the fit of their optimal model. For each top gene, the parameter estimates of their optimal model are used to fully characterize the gene’s expression dynamics across the time-course. A global summary of the dynamic changes across all top genes is then represented by the distribution of breakpoints across all time points. Our method does not require replicate time points and although we focus on time-course of gene expression, it may be applied to alternative features (e.g. isoform or micro-RNA expression) and/or other experiments with ordered conditions (e.g. spatial course).

## Implementation

Trendy is written in R and freely available on Bioconductor at https://bioconductor.org/packages/release/bioc/html/Trendy.html

**Fig. 1:**
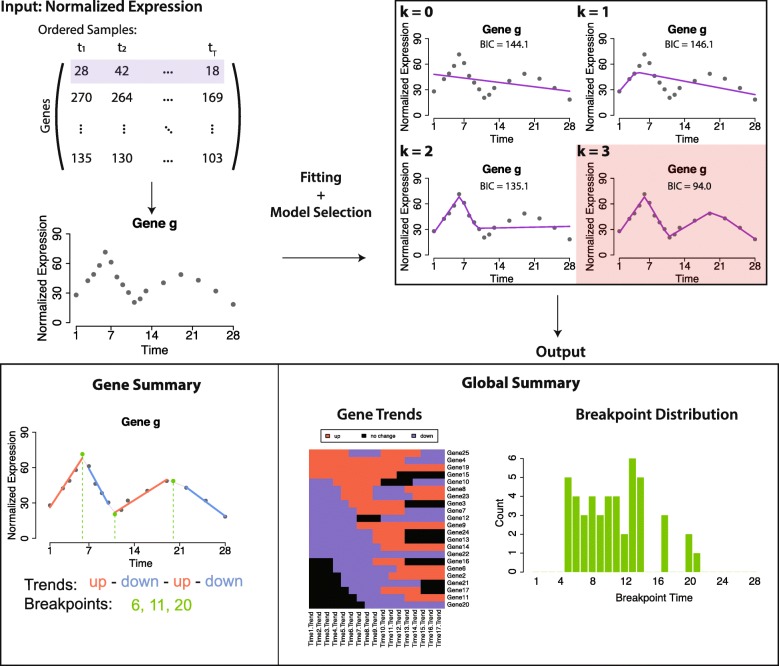
Trendy framework. The Trendy framework fits multiple segmented regression models to each feature/gene. The optimal model is selected as the one with the smallest BIC. Trendy summarizes the expression pattern of each gene and provides a summary of global dynamics

We include a detailed vignette with working examples and an R/Shiny application to visualize and explore the fitted trends. An overview of the Trendy framework is given in Fig. 1 and details on the implementation are given below.

### Input

The input data should be a *G* - by - *N* matrix containing the normalized expression values for each gene and each sample. Between-sample normalization is required prior to Trendy, and should be performed according to the type of data (e.g. Median-Normalization [12] for RNA-seq data or RMA [13] for microarray data). The samples should be sorted following the time-course order. A time vector should also be supplied to denote the relative timing of each sample. This is used to specify the spacing of time points and indicate any replicated time points. The user should also specify the total number of breakpoints considered per gene. The default value is *K*=3, but may be specified via the parameter maxK.

### Model fitting

We denote the normalized gene expression of gene *g* and sample/time *t* as *Y*_*g*,*t*_ for a total of *G* genes and *N* samples. We directly model *Y*_*g*,*t*_ as a function of time *t*, where *t*∈*t*_1_,…,*t*_*T*_, if time points are not replicated then *N*=*T*, otherwise for replicated experiments *N*≥*T*. The model for gene *g* with *k* breakpoints is:

1 $$ {\begin{aligned} {}M^{k}_{g}: Y_{g} \sim \beta^{k}_{g,0} + & \beta^{k}_{g,1}*t*I \left\{t: t\ge t_{1},t \le b^{k}_{g,1}\right\} + \ldots \\ + & \beta^{k}_{g,k+1}*\left(t-b^{k}_{g,k}\right)*I \left\{t: t\ge b^{k}_{g,k}+1, t \le t_{T}\right\} \end{aligned}}  $$

We aim to estimate *k* breakpoints, $b^{k}_{g,1}, b^{k}_{g,2}, \ldots, b^{k}_{g,k}$, occurring between *t*_1_ and *t*_*T*_. We also estimate *k*+2*β*’s: $\beta ^{k}_{0}$ indicates the intercept and the remaining *k*+1*β*’s indicate slopes for the *k*+1 segments separated by *k* breakpoints. Estimation of the model parameters is done using the iterative method in Muggeo, 2003, a key of which is linearizing the segmented regression model in (1) [14]. The method is available via the segmented R package [15].

### Model selection

For each gene, we fit *K*+1 models for *k*∈{0,1,…,*K*} and select the model having the optimal number of breakpoints by comparing the Bayesian information criterion (BIC) [16] among all models:

$${}{\begin{aligned} \tilde{k}_{g} = \text{argmin}_{k = 0,...,K} \text{BIC}_{g,k} = \text{argmin}_{k = 0,...,K} log(N)(2k+3) - 2\hat{L}_{M^{k}_{g}} \end{aligned}} $$

where $\hat {L}_{M^{k}_{g}}$ denotes the log-likelihood for the segmented regression model with *k* breakpoints for gene *g*. For a model with *k* breakpoints, there are *k* estimated breakpoints, *k*+1 estimated segment slopes, and an estimated intercept and error. The BIC of the linear model having no breakpoints, *k*=0, is also considered here.

### Goodness of Fit

An optimal model will be found for every gene, however we only further consider those genes with a good fit. We quantify the quality of the fit for each gene’s optimal model as the adjusted *R*^2^, which also penalizes for model complexity, as:

$$\bar{R}^{2}_{g,\tilde{k}_{g}} = 1 - \left(1 - R^{2}_{g,\tilde{k}_{g}}\right)\frac{N-1}{N-\left(\tilde{k}_{g}+1\right)-1}$$

where $\tilde {k}_{g}$ represents the optimally chosen *k* for gene *g*.

### Output

Trendy reports the following for each gene’s optimal model:

- Gene specific adjusted *R*^2^: $\bar {R}^{2}_{g,\tilde {k}_{g}}$
- Segment slopes: $\beta ^{\tilde {k}_{g}}_{g,0},\ldots, \beta ^{\tilde {k}_{g}}_{g,\tilde {k}_{g}+1}$
- Breakpoint estimates: $b^{\tilde {k}_{g}}_{g,1},\ldots, b^{\tilde {k}_{g}}_{g,\tilde {k}_{g}}$

To avoid overfitting, the optimal number of breakpoints will be set as $\tilde {k}_{g} = \tilde {k}_{g} - 1$ if at least one segment contains less than mNS data points. The threshold mNS can be specified by the user via the minNumInSeg argument; the default is five. Trendy characterizes expression patterns for only the top dynamic genes, defined as those whose optimal model has high $\bar {R}^{2}_{g,\tilde {k}_{g}}$. The default cutoff is.5, but may be specified by the user.

Trendy also summarizes the fitted trend or expression pattern of top genes. Once the optimal model for a gene is selected, each segment is assigned a direction of ‘up’, ‘down’, or ‘no-change’ based on the sign and p-value of its slope estimate $\beta ^{\tilde {k}_{g}}_{g,i}$. The p-value is obtained by comparing the t-statistic calculated from the slope coefficient and its standard error to the t-distribution with one degree of freedom. If the p-value is greater than *c*_*pval*_ the trend of the segment will be defined as ‘no-change’, otherwise, if the p-value is smaller than *c*_*pval*_ the segment is set to ‘up’ or ‘down’ depending on the sign of the slope. The default value of *c*_*pval*_ is 0.1, but may be specified by the user. Trendy represents the trends ‘up’, ‘down’, and ‘no-change’ as 1, -1, and 0, and genes fitted trends may be clustered using an algorithm such as hierarchical clustering. Genes in the same group may then be investigated using gene enrichment analysis [17–19] to examine whether common functional annotations exist.

A global view of expression changes is obtained by computing the breakpoint distribution as the sum of all breakpoints at each time point over all dynamic genes:

$$D_{t} = \sum_{g = 1,...,G} \sum_{i = 1,...,\tilde{k}_{g}} I \left\{b^{\tilde{k}_{g}}_{g,i} = t \right\}$$

### Visualization

The Trendy package includes an R/Shiny application which provides visualization of gene expression and the segmented regression fit. The application also allows users to extract a list of genes which follow particular expression patterns. The interface is shown in Additional file 1: Figure S1.

## Results

### Simulation study

We performed a simulation study to illustrate the operating characteristics of Trendy using an RNA-seq dataset with *N*=96 samples. The data are technical replicates collected and sequenced at the same time following the protocol from Hou et al., 2015 [20], and thus have no expected trend (this dataset is provided here as Additional file 2). Data were simulated through repeatedly shuffling the sample order of this dataset and assigning time points.

We investigated the effect of the following parameters on the number of dynamic genes identified by Trendy:

- Total number of breakpoints: *K*=1,5,10.
- Minimum number of time points required in a segment: mNS=2,5.
- Total length of time course: *T*=25,50.
- Distribution of time points:
  - Evenly spaced and short (*t*={1,2,…,24,25}).
  - Evenly spaced and long (*t*={1,5,…,120,125}).
  - Randomly spaced (*t*_*i*_ sampled from{1,2,…,124,125} without replacement).

Each combination of the parameter settings described above were used to evaluate Trendy over 100 independent simulations. After removing genes with zero expression in all samples prior to the simulation, the number of genes remaining was *G*=16,862. Trendy additionally filters genes below a given mean expression, and here the default cutoff of 5 was used, which left approximately *G*∼10,000 in each simulation, varying slightly depending on the subset of samples included. For each scenario, the number of false positives was defined as the number of top dynamic genes in two ways: those with $\bar {R}^{2}_{g,\tilde {k}_{g}} >.5$ or $\bar {R}^{2}_{g,\tilde {k}_{g}} >.8$. Ideally, Trendy should identify zero genes with dynamic trends for these simulated datasets.

**Fig. 2:**
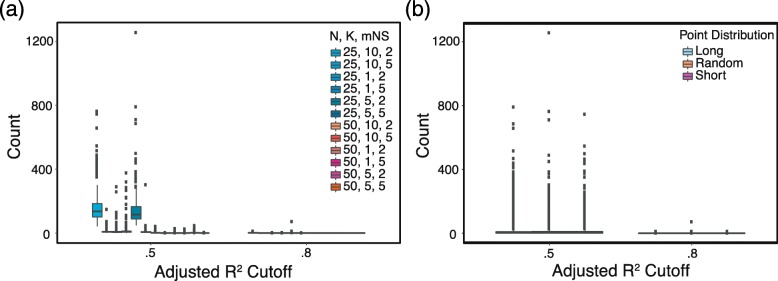
Simulation results. A set of replicate RNA-seq samples collected at the same time were shuffled and assigned a time-order. The number of top dynamic genes identified by Trendy was determined using two adjusted *R*^2^ thresholds of $\bar {R}^{2}_{g,\tilde {k}_{g}} >.5$ and $\bar {R}^{2}_{g,\tilde {k}_{g}} >.8$. Shown in panel **a** are the number of genes above the $\bar {R}^{2}_{g,\tilde {k}_{g}}$ cutoffs for all combinations of settings for *K*,*N*,and mNS (each combination was simulated 300 times over varying point distributions). Panel **b** contains the number of genes above each cutoff over three various point distribution scenarios (each box contains 2400 simulations over all other varied parameters)

As shown in Fig. 2, Trendy generally identified few false positives even with a threshold of.5. Over all scenarios, an average of 30 genes (median = 3) had $\bar {R}^{2}_{g,\tilde {k}_{g}} >.5$, while an average of 0 (median = 0) genes had $\bar {R}^{2}_{g,\tilde {k}_{g}} >.8$. Figure 2a separates scenarios based on each combination of *N*,*K*,and mNS. Two scenarios produced the largest number of dynamic genes identified, these were *N*=25,*K*=10,mNS=2 and *N*=25,*K*=5,mNS=2, with an average of 163 and 149 identified genes with $\bar {R}^{2}_{g,\tilde {k}_{g}} >.5$, respectively. These two settings only require two data points separating potential breakpoints. Figure 2b demonstrates that there was no difference in the number of genes detected across variations of the time point distribution.

An additional simulation study was performed to illustrate the operating characteristics of Trendy when true trends are present in the data. We simulated each gene to have between zero and two breakpoints and the slope of each segment was randomly simulated as ‘up’, ‘down’ or ‘no-change’. In order to evaluate Trendy’s performance with differing variances, all genes are simulated to have the same mean. Each time point was simulated to have three replicates with biological variability matching that of the Axolotl dataset (described below in Application to RNA-seq data).

Specifically, the variability settings were:

- Low: Variances sampled from the 20–30th percentile of variability.
- High: Variances sampled from the 70–80th percentile of variability.

These two settings were simulated 100 times with *G*=50 and *N*=25. We used default settings when variance is low, and for high variance the p-value cutoff, *c*_*pval*_, was set to.2. We evaluated the performance of Trendy based on the average percentage of genes correctly classified in the number of breakpoints, trend, and the time of breakpoints (when applicable). The full results are shown in Table 1. Overall, Trendy identified the correct number of breakpoints for 97% of genes when variance is low and 90% with high variance. The trend is correctly identified for 93% and 84% of genes when variance is low and high, respectively. Gene trends that were misclassified were largely ones initially simulated as either ‘up’ or ‘down’, but appeared closer to ‘no-change’ once the variability was added. This also accounts for the observed decrease in trend classification as *K* increased for this simulation.

**Table 1:** Results of simulation study for genes having a true simulated trend

	Low variance		High variance	
Average % correct:	K	Trend	K	Trend
K = 0	100%	99%	100%	94%
K = 1	97%	93%	92%	86%
K = 2	95%	88%	79%	72%

The average percent of genes over all simulations classified correctly in terms of K and the trend direction when the true K is simulated as either 0, 1, or 2 and the within-gene variance is either low or high

For genes that Trendy correctly estimated the number of breakpoints, we evaluated the estimation of breakpoint time. Specifically, we calculated the deviation of the estimated breakpoints to the true simulated value when *K*=1 or *K*=2. The estimated breakpoint time was highly accurate, with an average difference of.01 when both *K*=1 and *K*=2 when variability was low and for the high variability scenario, the average difference was zero when *K*=1 and -.01 when *K*=2.

### Time of computation

The computation time of Trendy scales approximately linearly in number of genes (*G*), number of samples (*N*), and number of breakpoints considered (*K*). On a Linux machine using 10 cores, Trendy takes approximately 3.4 h for a dataset with 10,000 genes, 30 time points, and with *K*=3.

### Applications

#### Application to microarray data

**Fig. 3:**
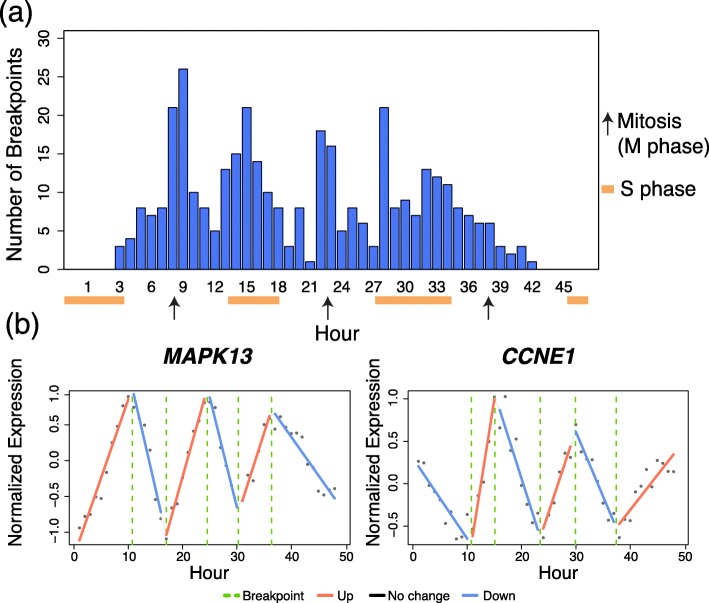
Results of Trendy on the Whitfield dataset. Panel **a** is the breakpoint distribution for the 118 genes having $\bar {R}^{2}_{g,\tilde {k}_{g}} >.8$. Orange bars indicate the S phase and black arrows indicate the time of mitosis as shown in Figures 1 and 2 in Whitfield et al., 2002. Panel **b** contains two genes identified by Trendy with different expression dynamics over the time-course

We applied Trendy to a microarray time-course dataset from Whitfield et al., 2002 [21]. In the Whitfield data, HeLa cells were synchronized and collected periodically for a total of 48 measured time points. Trendy identified a total of 118 top genes, defined as those having $\bar {R}^{2}_{g,\tilde {k}_{g}} >.8$. Figure 3a shows the total number of breakpoints over time for all top genes. The hours with the most breaks/changes in gene expression directly correspond to times of mitosis and completion of the cell cycle as described in Fig. 1 of Whitfield et al., 2002. Figure 3b shows two genes with fitted models from Trendy having different dynamic patterns. Both genes have 5 estimated breakpoints, however the first gene, *MAPK13* peaks at hours 9, 22, and 34. The second gene, CCNE1, peaks at hours 14 and 28. These peak times also correspond to the cell cycle stages since *CCNE1* is active during G1/S transition and *MAPK13* is most active during the M phase.

Further analysis by Trendy identified a total of 34 top genes that have a cycling pattern defined as “up-down-up-down” (Additional file 1: Figure S2). Of these genes, 20 are directly annotated to the Gene Ontology (GO) [22] cell cycle pathway (GO:0007049), while others are linked to related activities such as DNA replication and chromosome organization. All but two genes were annotated to the cell cycle in the original publication; both genes (*HBP* and *L2DTL*) are now supported in the literature as being involved in the cell cycle.

#### Application to RNA-seq data

**Fig. 4:**
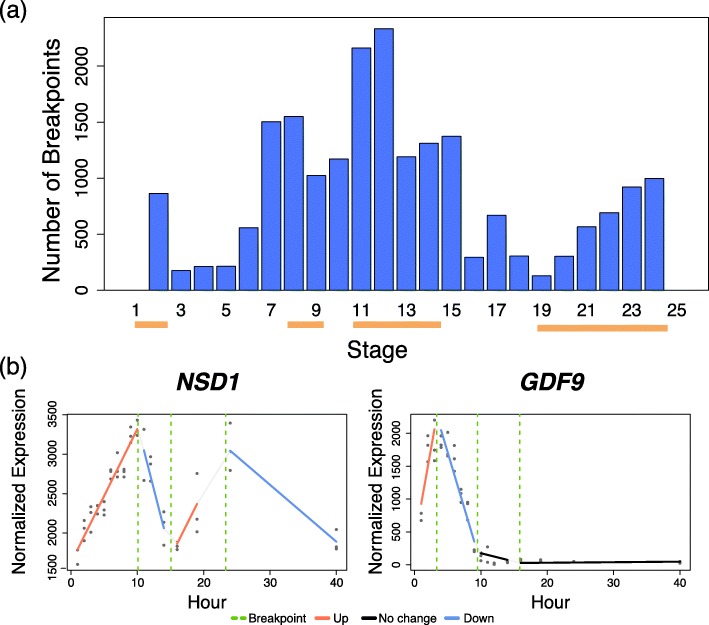
Results of Trendy on the Axolotl dataset. Panel **a** contains the breakpoint distribution for all 9535 genes having $\bar {R}^{2}_{g,\tilde {k}_{g}} >.8$. The orange bars indicate the times of major transcriptome changes identified in Figure 2 in Jiang and Nelson et al., 2016. Panel **b** shows two genes identified by Trendy with different expression dynamics over the time-course. The first gene, *NSD1*, has three estimated breakpoints, while *GDF9* has two breakpoints

We applied Trendy to the full RNA-seq time-course dataset from Jiang and Nelson et al., 2016 [7] which examined axolotl embryonic development. In the axolotl data, embryos were collected at distinct developmental stages representing specific development milestones. RNA-seq was performed consecutively for Stage 1 through Stage 12, and then periodically until Stage 40 for a total of 17 stages measured. Trendy identified a total of 9535 genes with $\bar {R}^{2}_{g,\tilde {k}_{g}} >.8$. Figure 4a shows the number of breakpoints over the developmental stages and Fig. 4b shows two genes with fitted models from Trendy having different dynamic patterns. In general, time periods where Trendy discovered a high number of breakpoints correspond to the waves of transcriptional upheaval as discovered by Jiang and Nelson et al., 2016.

Further analysis by Trendy identified 807 genes having a delayed peak pattern defined as “same-up-down” with the first breakpoint occurring after Stage 8 (Additional file 1: Figure S3). Enrichment analysis of the genes was performed based on gene-set overlaps in MSigDB (v6.0 MSigDB, FDR q-value <.001, http://software.broadinstitute.org/gsea/msigdb) [23, 24]. The top 10 categories of enriched GO biological processes (GO [22]) include embryo development (GO:0009790), regulation of transcription (GO:0006357), organ/embryo morphogenesis (GO:0009887), tissue development (GO:0009888), regionalization (GO:0003002), and pattern specification (GO:0007389). These categories closely match those identified in the original publication [7]. Genes which contain at least two peaks and appear to have cyclic activity contain enrichments for chromosome organization (GO:0051276) and regulation of gene expression (GO:0010629) within the top ten categories. The full set of enrichment results are given in Additional file 3.

Trendy was also applied to two neural differentiation time-course RNA-seq experiments in Barry et al. 2017 [6]. Breakpoints were estimated separately for the mouse and human differentiation time-course experiments and peaking genes were identified as those having the pattern “up-down”. The authors there found that the relationship between mouse and human peak-times estimated using Trendy for top ranked neural genes closely matched that expected by the gold-standard Carnegie stage progressions [6].

### Comparison to other methods

To highlight the main differences between Trendy and other tools such as EBSeq-HMM and FunPat, we performed a comparative study using the Axolotl RNA-seq dataset. The dataset has 17 measured time points with 2 or 3 replicates at each time. Because EBSeq-HMM attempts to classify genes into 3^time points−1^ patterns, it is not computationally tractable for very long time-courses and we were not able to run the method on this set of data.

FunPat is able to analyze datasets with a large number of time points, however the output is different from that of Trendy in a number of ways. Since there is no standard annotation package in R for axolotl genes, we focus on the output of the gene clustering. FunPat identified 411 total patterns using the default settings. The patterns are represented visually for each group and a text file lists the genes belonging to each cluster as well as standardized expression values for each gene. Additional file 1: Figure S4 contains an example of a gene cluster identified by FunPat and the Trendy fit for a selected set of genes. We find that individual gene patterns and times of expression changes appear to vary noticeably within FunPat clusters. Also, the total run time for FunPat was 11 h on an 8-core Mac desktop with 16 GB RAM. In comparison, the total run-time for Trendy was 1.5 h.

**Fig. 5:**
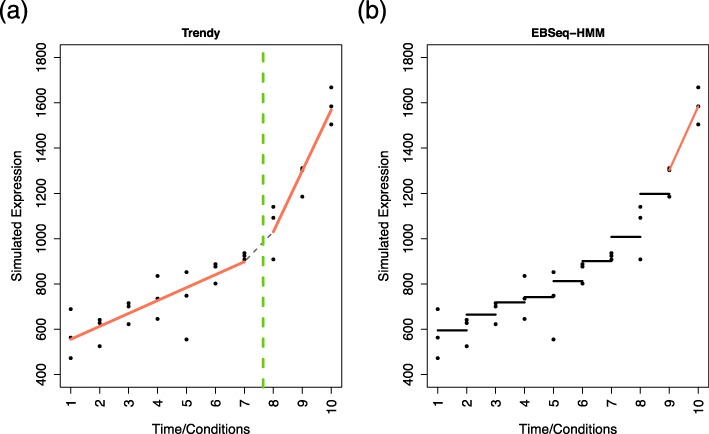
Comparison to EBSeq-HMM. The reported expression trend for a single simulated gene analyzed using Trendy and EBSeq-HMM is shown. In **a** Trendy reports two increasing segments separated by a breakpoint between times 7 and 8. In **b** EBSeq-HMM reports the expression path as “EE-EE-EE-EE-EE-EE-EE-EE-Up”, where ‘EE’ is equivalent to ‘no-change’

To illustrate an example of Trendy versus EBSeq-HMM on a shorter time-course dataset, we demonstrate one simulated gene example in Fig. 5. This gene is generated from the simulation study set-up with N = 10, high variance, *K*=1, and an increasing trend over the time-course. In Fig. 5a, Trendy correctly characterizes this gene as having pattern “up-up”. The two segments have different “up” magnitudes and a breakpoint is correctly detected between times 7 and 8. EBSeq-HMM was run with the expected fold change value lowered to 1.5 and otherwise default settings. Figure 5b shows that EBSeq-HMM classifies this gene’s pattern as “EE-EE-EE-EE-EE-EE-EE-EE-Up” with posterior probability.5, where ‘EE’ is equivalent to ‘no-change’.

## Discussion

We developed an approach we call Trendy, which utilizes segmented regression models to analyze data from high-throughput expression profiling experiments with ordered conditions. Trendy provides statistical analyses and summaries of both feature-specific and global expression dynamics. In addition to the standard workflow in Trendy, also included in the R package is an R/Shiny application to visualize and explore expression dynamics for specific genes and the ability to extract genes containing user-defined patterns of expression.

Trendy characterizes genes more appropriately than EBSeq-HMM for long time-courses when the expression is noisy and changes are gradual over the time-course. Although an alternative auto-regressive model for EBSeq-HMM might provide the flexibility to better classify genes in such cases, we also stress that Trendy provides unique information on dynamics including the time of significant changes via the breakpoint estimation. Trendy is also able to handle much longer time-courses in a reasonable amount of time compared to EBSeq-HMM and FunPat. In addition, the output of Trendy is more flexible than FunPat as genes can be clustered based on a variety of summaries provided such as breakpoint location and trends.

Trendy performed well in both simulation studies by identifying few false positive genes when no trend was present and correctly identifying breakpoints and trend directions when true trends were simulated. As demonstrated in the simulations, Trendy is robust at choosing the true K. However, in practice, setting K much larger than what is biologically reasonable is not advised since it increases the computation time. We also note that the number of data points in a segment separating breakpoints, mNS, is a critical parameter. The choice of this parameter value is directly linked to the number of samples *N*. For example, if a time-course has *N*=*T*=10 then it is not possible to identify any breakpoints if mNS=10. Rather, a smaller number of data points separating the breakpoints would be required, such as mNS = 4, which would allow a maximum of one breakpoint to be fit and require at least 4 data points in both segments surrounding the breakpoint. Based on the simulations and case studies, mNS around five is recommended, which also indicates that Trendy is designed for experiments with *T*>10. In general, Trendy is intended for more densely sampled biological processes, where multiple time points carry evidence of a trend. If a significant change is expected between two consecutive time points that is not supported by the surrounding times and replicates are not available then EBSeq-HMM is more appropriate to assess statistical significance.

In addition to characterizing each gene, Trendy also calculates a global summary of dynamic changes. The breakpoint distribution can be used to prioritize follow up investigations or experiments into specific time points. We recommend using the top dynamic gene breakpoints to generate this by specifying those with a higher value of $\bar {R}^{2}_{g,\tilde {k}_{g}}$.

## Conclusion

We applied Trendy to two case study datasets (one microarray and one RNA-seq) and demonstrated the approach’s ability to capture biologically relevant information in individual gene estimates of breakpoints and trends, as well as, information conveyed in global summaries of trends across genes. Although Trendy was applied only to single-series time course experiments here, the breakpoints for Trendy can be compared across experiments if measured on the same time or spatial scale as we did in Barry et al., 2017. In experiments where the number of time points is large and/or expression between time points is consistent yet subtle, we expect Trendy to be a valuable tool, especially as the prevalence of such experiments is on the rise with an increase in time-course sequencing experiments to study dynamic biological processes and the proliferation of single-cell snapshot sequencing experiments in which cells can be computationally ordered and assigned a temporal (or spatial) order [25–27].

## Availability and requirements

**Project name:** Trendy

**Project home page:** https://bioconductor.org/packages/release/bioc/html/Trendy.html

**Operating system(s):** all, specifically tested on Linux and Mac

**Programming language:** R

**Other requirements:** R version ≥3.4

**License:** GPL-3

**Any restrictions to use by non-acadecmics:** No restrictions.

## Additional files

Additional file 1 Supplementary Figures. (PDF 3581 kb)

Additional file 2 RNA-seq data used in simulation study. (TXT 10,180 kb)

Additional file 3 Enrichment results of Axolotl RNA-seq data. (XLSX 105 kb)

## Abbreviations

RNA-seq: RNA sequencing BIC: Bayesian information criterion GO: Gene Ontology
