# Supplementary material for: Trendy: segmented regression analysis of expression dynamics in high-throughput ordered profiling experiments
Source: BMC Bioinformatics. 2018 Oct 16;19:380. doi: 10.1186/s12859-018-2405-x (PMC6192113; doi:10.1186/s12859-018-2405-x)
Supplement: Supplementary file 1 — Supplementary Figures. (PDF 3581 kb) [file 12859_2018_2405_MOESM1_ESM.pdf]

Supplementary Figures

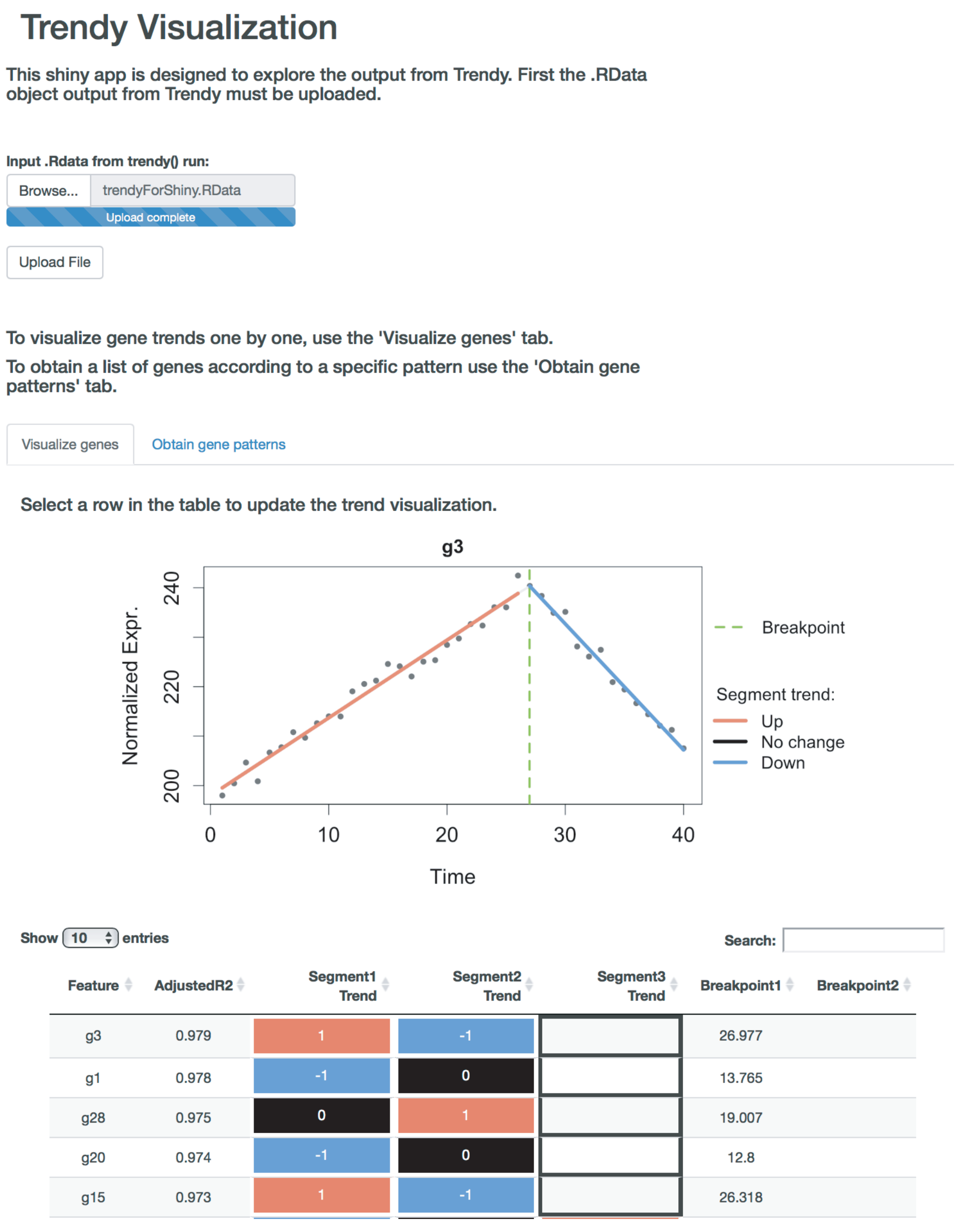

Figure S1: Screenshot of Trendy R/Shiny application The trendy() function outputs an .RData object which can be uploaded to the Trendy R/Shiny application available within the Trendy R package via the function trendyShiny(). Users can explore the data, fitted trends, and easily extract lists of genes with specific patterns of interest.

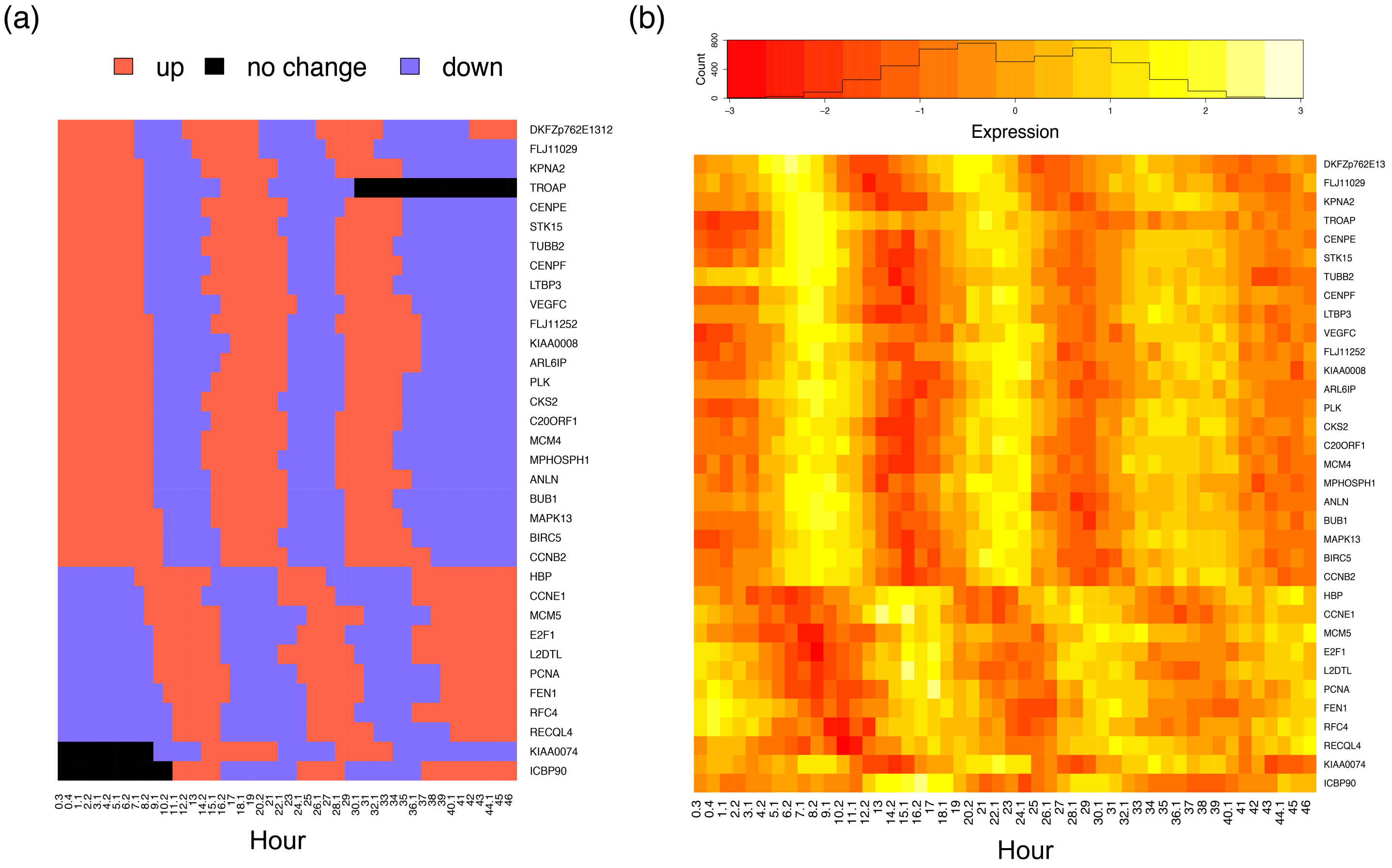

Figure S2: Expression dynamics for cycling genes in Whitfield dataset Panel (a) shows the fitted trends for the 34 top genes having pattern "up-down-up-down" and Panel (b) is an expression heatmap for the same set of genes.

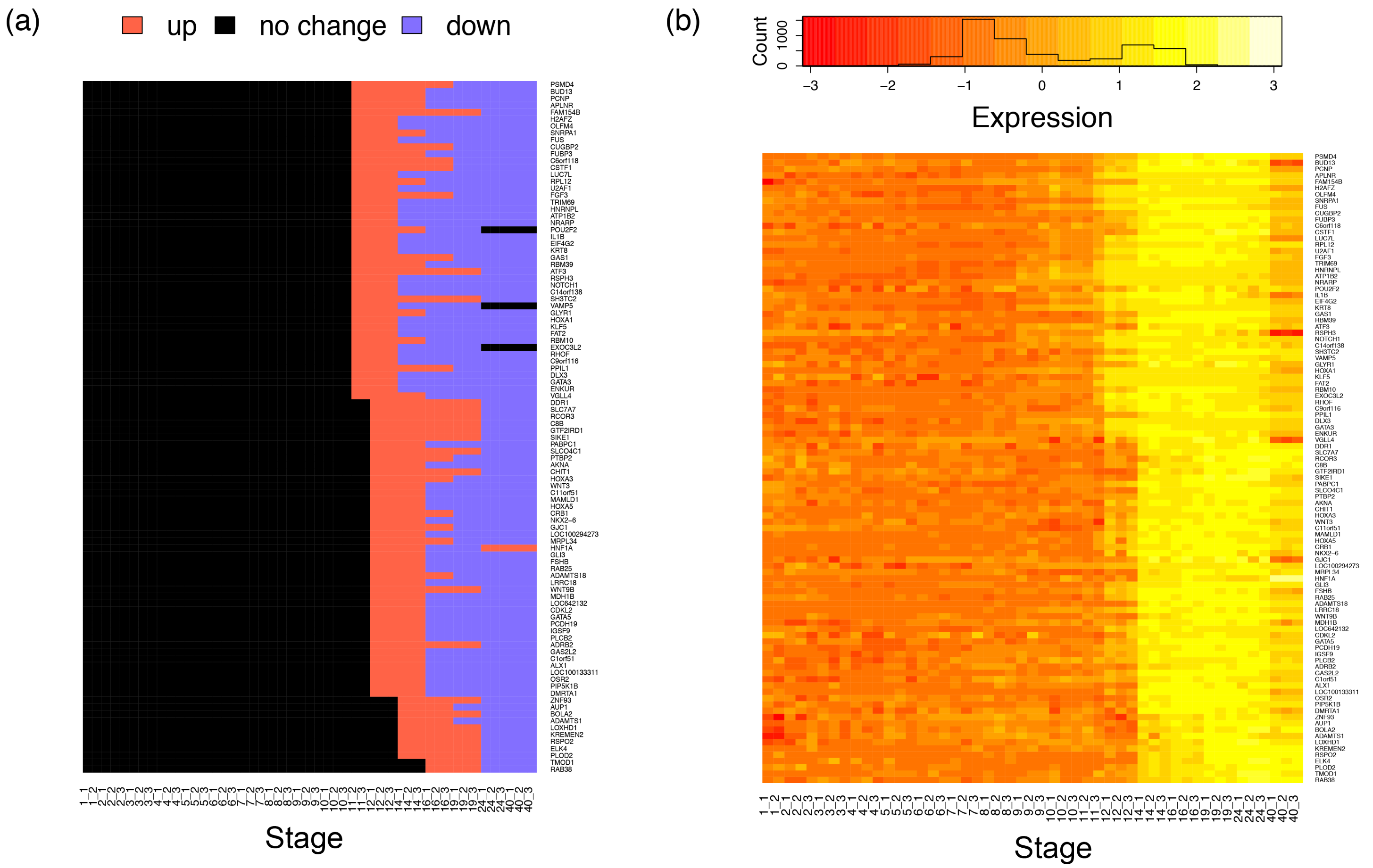

Figure S3: Expression dynamics for delayed peak genes in the Axolotl dataset (a) Fitted trends and (b) expression heatmaps are shown for a subset of 100 top genes having pattern "same-up-down" with breakpoint after Stage 8.

FunPat Output Example:

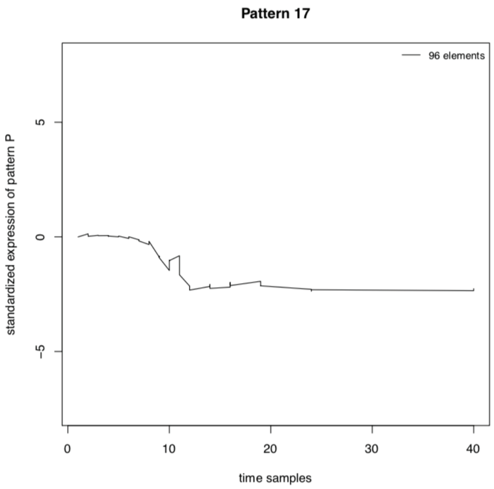

Element_ID	Score	Cluster	t1	t2
MTMR6	8.25E-05	17	-0.002788929	0.131623627
MPPED2	9.91E-05	17	-0.002788929	0.131623627
ZNF347	0.00076847	17	-0.002788929	0.131623627

Trendy Output Example:

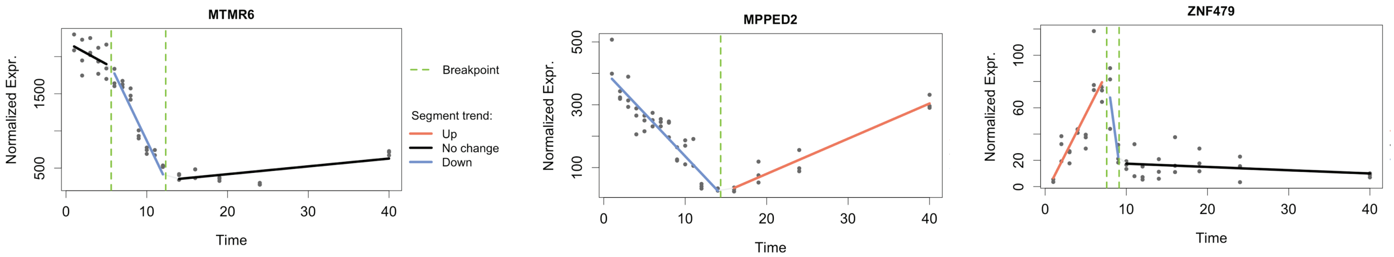

Figure S4: Comparison to FunPat The output of FunPat for one example cluster is shown. Specifically, Pattern 17 found in the Axolotl data contains a total of 96 genes. Three of those genes are shown with their respective Trendy fits.
